# Performance of imaging studies in patients with suspected appendicitis after stratification with adult appendicitis score

**DOI:** 10.1186/s13017-017-0119-4

**Published:** 2017-01-31

**Authors:** Henna E. Sammalkorpi, Ari Leppäniemi, Eila Lantto, Panu Mentula

**Affiliations:** 1 Department of Gastrointestinal Surgery, Helsinki University Central Hospital, Helsinki, Finland; 2 University of Helsinki, Medical Faculty, Helsinki, Finland; 3 Department of Radiology, Helsinki University Central Hospital, Helsinki, Finland

**Keywords:** Appendicitis, Imaging, diagnostic, Abdomen, acute, Adult, Ultrasonography, diagnostic, Multidetector computed tomography

## Abstract

**Background:**

Diagnostic scoring is used to stratify patients with suspected appendicitis into three groups: high, intermediate, and low probability of appendicitis. The stratification can be used for selective imaging to avoid the harms of radiation without compromising diagnostic accuracy.

The aim was to study how stratification by Adult Appendicitis Score affects diagnostic performance of imaging studies.

**Methods:**

Analysis of 822 patients who underwent diagnostic imaging for suspected appendicitis was made. Adult Appendicitis Score was used to stratify patients into groups of high, intermediate, and low probability of appendicitis. Diagnostic performance of computed tomography (CT) and ultrasound (US) was compared between these patient groups.

**Results:**

After scoring, pre-test probability of appendicitis ranged from 9-16% in low probability group to 75-79% in high probability group in patients who underwent US or CT. Post-test probability of appendicitis after positive CT was 99, 91, and 75% in high probability, intermediate probability and low probability groups, respectively, *p* < 0.001. After positive US the respective probabilities were 95, 91 and 42%, *p* < 0.001.

**Conclusion:**

Diagnostic imaging has limited value in patients with low probability of appendicitis according to Adult Appendicitis Score.

## Background

CT and US are practical tools in diagnosis of acute appendicitis [1–3]. Lack of guidelines regarding the diagnostic use of imaging may, however, lead to either under- or overuse of these imaging modalities. In many institutions, imaging is mandatory in suspected acute appendicitis [4–6]. Routine CT on all patients with suspected appendicitis induces risks of ionizing radiation and contrast medium as well as increased delay to correct diagnosis and treatment [7–11]. US involves no ionizing radiation, but there is great variance in reported diagnostic performance. The reported sensitivity ranged from 44 to 100% and specificity from 47-99% in a meta-analysis [12]. The aim of avoiding excess radiation has, with good outcomes, led to US utilization as a screening method with additional CT in case of negative or inconclusive finding [1, 4, 6].

In a meta-analysis by van Randen et al. the prevalence of appendicitis was reported to influence the sensitivity and specificity of imaging and benefit less in patient groups with the highest and lowest probabilities of appendicitis [13]. Nevertheless, mandatory imaging for all patients with right lower quadrant abdominal pain is common.

**Fig. 1:**
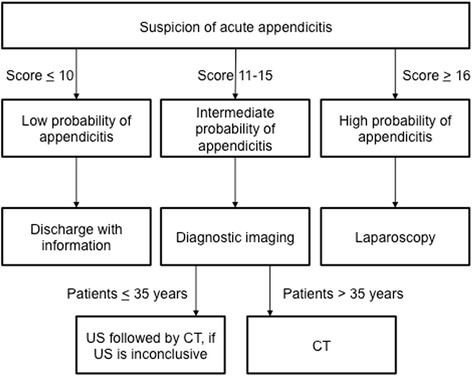
Diagnostic work-up of suspected acute appendicitis with Adult Appendicitis Score (AAS). MRI was performed instead of CT in pregnant patients

Diagnostic scoring is a simple, free and fast method for stratifying patients according to risk of appendicitis [14, 15]. Diagnostic scoring is recommended in EAES 2015 consensus guidelines and WSES 2016 guidelines as a part of diagnostic algorithm for suspected appendicitis [16, 17]. Because of somewhat insufficient discriminating capacities of existing scoring systems, we constructed a novel scoring system, Adult Appendicitis Score (AAS) [18]. The score stratifies patients with suspected appendicitis in three groups according to probability of appendicitis: high, intermediate, and low probability. Instead of replacing imaging, AAS helps to accurately select patients with most uncertain diagnosis to imaging. (Table 1, Fig. 1, www.appendicitisscore.com) Adult Appendicitis Score has been validated and it is now in our hospital part of routine diagnostic work-up of patients suspected of acute appendicitis. In the validation study specificity and sensitivity of high-probability group of the new score were 93.3 and 49.4%, respectively. The negative predictive value of AAS (likelihood of no appendicitis in the low-risk group) was 93% [19].

**Table 1:** Adult Appendicitis Score

Symptoms and findings		Score
Pain in RLQ		2
Pain relocation		2
RLQ tenderness	Women, age 16-49	1
	All other patients	3
Guarding	mild	2
	moderate or severe	4
Laboratory tests		
Blood leukocyte count (x10^9^)	> = 7.2 and <10.9	1
	> = 10.9 and <14.0	2
	> = 14.0	3
Proportion of neutrophils (%)	> = 62 and < 75	2
	> = 75 and < 83	3
	> = 83	4
CRP (mg/l), symptoms < 24 h	> = 4 and <11	2
	> = 11and <25	3
	> = 25 and <83	5
	> = 83	1
CRP (mg/l), symptoms > 24 h	> = 12 and <53	2
	> = 53 and <152	2
	> = 152	1

*RLQ*, right lower abdominal quadrant

Diagnostic performance of imaging has not been compared between patient groups of different probability of appendicitis stratified by diagnostic score. Because of potentially high frequency of false positive imaging results, mandatory imaging can induce negative appendectomies in patients with low probability of appendicitis.

This study aimed at evaluating the diagnostic performance of CT and US in patients with different pre-imaging probabilities of appendicitis stratified by Adult Appendicitis Score.

## Methods

### Patients

We performed an analysis of prospectively collected data of adult (≥16 years) patients at the emergency department. The data were collected in two periods (2011 and 2014–2015). All patients with acute right lower abdominal quadrant pain and/or suspected acute appendicitis were included in the original data collection. For the current study all patients that underwent diagnostic imaging for suspected appendicitis were included. The first data collection was originally for the construction, and the second for the validation of the new diagnostic score. Patients and methods for the first data collection are described in more detailed fashion in the original article of the construction of the score [18]. During the first data collection there were no guidelines of diagnostic work-up of patients with suspected acute appendicitis. Imaging was at all times available and performed at each surgeon’s discretion.

In the beginning of the second study period, the AAS was introduced into emergency room routine to guide the diagnostic work-up of patients suspected of acute appendicitis. With the help of AAS patients were stratified in three groups of different probabilities for appendicitis - high, intermediate and low probability. A recommendation according to scoring was provided as follows: High-probability patients could be operated on without further examinations whereas low-probability patients could be discharged. Patients in the intermediate-probability group should undergo diagnostic imaging. This way diagnostic scoring, instead of replacing imaging, helps to accurately select patients with most uncertain diagnosis to imaging [18]. Scoring was performed with a web application that calculated the score and suggested further action based on the scoring result. (Figure 1) Scoring was mandatory, but adherence to the associated guidelines was not controlled. Each surgeon responsible for the patient was able to perform diagnostic imaging regardless of the scoring result.

Both data collections were performed at the emergency department by the surgeons on duty. Additional data was retrieved from patient databases. The collected data included all variables required for scoring, patient demographics, results of possible diagnostic imaging, surgery, histological analysis of appendix, final diagnosis, timing of surgery, delay to diagnosis and surgery, and possible complications. The patients’ medical records were reviewed after a minimum of one month after hospital discharge for possible misdiagnosis and complications.

At surgeries for suspected appendicitis, the appendix was at all times removed, and the final diagnosis of appendicitis was invariably based on histological analysis showing transmural neutrophilic inflammation of appendix.

### Imaging procedures

In patients of age 35 or less and all pregnant patients, US was recommended as a primary imaging modality, CT (or MRI in pregnant patients) was recommended in case of negative or inconclusive US.

US examinations were performed by radiology residents with minimum experience of 2 years or attending radiologists with a possibility to consult a more experienced colleague. A general survey of the abdomen and pelvis was done using the graded compression technique with convex 3.5 – 5 MHz probe and linear 6–12 MHz probe (GE Logic 9E, GE Healthcare, Wisconsin, USA). Inconclusive US reports were classified as negative for appendicitis in this study.

CT scans were performed by using 128 multi-detector row scanner with automatic tube current and tube voltage modulation (Somatom Definition AS+, Siemens Medical Systems, Erlangen, Germany). Patients underwent an abdominopelvic CT protocol with intravenous contrast-enhancement (iohexol, Omnipaque 350 mgI/ml, GE Healthcare, Oslo, Norway, bolus 1,5 ml/kg body weight at 3 ml/s flow rate) in portal venous phase. Patients with known renal failure or hypersensitivity to contrast media underwent unenhanced CT. CT parameters were as follows: reference mAs 110, reference kV 120, collimation 128 x 0,6 mm, rotation time 0,5 s. Data was reconstructed at 3 mm axial, coronal and sagittal slices and analysed using PACS workstations by a staff radiologist during working hours and by a radiologic resident after hours. These original reports contributing to surgeons’ decision-making were used in study analysis. Effective dose of low dose CT was 3.2 mSv in women and 2.6 mSv in men.

Non-compressible appendix larger than 6 mm in diameter with or without appendicolith together with local transducer tenderness, and peri-appendiceal fat infiltration were criterion for acute appendicitis in ultrasound.

On CT, increased appendiceal diameter (greater than 6 mm), with or without appendicolith together with appendiceal wall thickening, increased wall enhancement, and peri-appendiceal fat infiltration were criteria for acute appendicitis.

### Statistical analysis

Statistical analysis was performed using SPSS® version 22 (IBM, Armonk, New York, USA). AAS was calculated for all patients. The pre-test probability (probability of appendicitis in patients undergoing imaging) and post-test probabilities (probability of appendicitis in patients with positive or negative imaging result) of acute appendicitis as well as accuracy, specificity, sensitivity, likelihood ratios, and diagnostic odds ratio for US and CT were calculated. Diagnostic performance of MRI was left outside further analysis because of small amount of patients.

These results were compared between patient groups of different prevalence of acute appendicitis stratified by AAS.

## Results

### All patients

Diagnostic imaging was performed on 822 (53%) of 1545 patients with suspected acute appendicitis. 892 (58%) of 1545 patients with suspected appendicitis had appendectomy, out of which 121 (13.6%) were not inflamed. Of all patients that underwent diagnostic imaging, 368 (45%) had appendicitis. CT was performed to 489 (32%), US to 497 (32%), and magnetic resonance imaging (MRI) to 14 (1%) patients. (Table 2).

**Table 2:** Prevalence of appendicitis in patients that underwent either no diagnostic imaging, US, CT or MRI

Probability of appendicitis	All patients^a^	No imaging^a^	CT^a^	US^a^	MRI^a^
All patients	724/1545 (46.9%)	356/723 (49.2%)	257/489 (52.6%)	177/497 (35.6%)	5/14 (35.7%)
High (AAS ≥16)	386/439 (87.9%)	261/282 (92.6%)	90/114 (78.9%)	41/52 (78.8%)	1/2 (50%)
Intermediate (AAS 11–15)	304/596 (51.0%)	89/172 (51.7%)	138/276 (50.0%)	122/258 (47.3%)	2/8 (25%)
Low (AAS ≤10)	34/510 (6.7%)	6/269 (2.2%)	16/99 (16.2%)	17/187 (9.1%)	2/4 (50%)

^a^Numbers show patients with appendicitis/total amount of patients in each group (%)

*AAS*, Adult Appendicitis Score

Pre-test probability of appendicitis in all patients that underwent CT was 257 of 489 (52.6%). The overall sensitivity and specificity of CT were 98.4 and 92.2%, respectively. The observed post-test probability for positive CT was 253 of 260 (97.3%) and for negative CT 4 of 229 (1.75%). The accuracy of CT (the proportion of correct (true positive or true negative) imaging results) 478 of 489 (97.8%).

Pre-test probability of appendicitis in all patients that underwent US was 177 of 497 (36.6%). The overall sensitivity and specificity of US were 48.6 and 94.4%, respectively. The post-test probability for positive US was 86 of 104 (82.7%), and for negative US 91 of 393 (23.2%). The overall accuracy of US was 388 of 497 (78.1%). (Tables 2-4).

### High probability group (AAS ≥16)

**Fig. 2:**
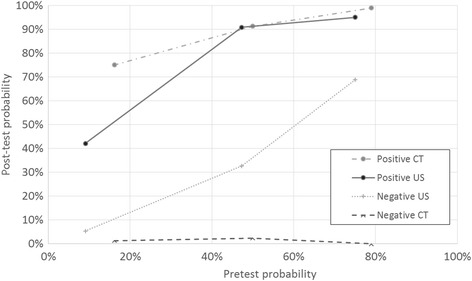
Pre-test and post-test probability of appendicitis after positive and negative imaging results. Accuracy of imaging was dependent on the pre-test probability of appendicitis. Negative CT was accurate in all patient groups

In the group of high probability of acute appendicitis there were 439 patients of whom 386 (88%) had appendicitis. CT was performed to 114 (26%) patients. In patients that underwent CT pre-test probability of acute appendicitis was 90 of 114 (78.9%). The post-test probability for appendicitis was for a positive test 90 of 91 (98.9%) and for a negative test 0 of 23 (0%). The accuracy of CT was in this group 113 of 114 (99.1%). (Table 3, Table 4, Fig. 2).

**Table 3:** Diagnostic performance of US and CT

Probability of appendicitis according to AAS	Sensitivity	Specificity	LR+	LR-	DOR
US					
All patients	48.6%	94.4%	8.646	0.545	15.86
High (AAS ≥16)	46.3%	90.9%	5.098	0.590	8.636
Intermediate (AAS 11–15)	48.4%	95.6%	10.971	0.540	20.291
Low (AAS ≤10)	47.1%	93.5%	7.274	0.566	12.848
CT					
All patients	98.4%	92.2%	12.615	0.017	742.06
High (AAS ≥16)	100.0%	95.8%	23.98	0	Infinite
Intermediate (AAS 11–15)	97.8%	90.6%	10.385	0.024	432.69
Low (AAS ≤10)	93.8%	94.0%	15.573	0.067	234.00

*AAS*, Adult Appendicitis Score, *LR+*, positive likelihood ratio, *LR*, negative likelihood ratio, DOR diagnostic odds ratio

**Table 4:** Pre- and post-test probabilities of appendicitis, patients who underwent US or CT

Probability of AA according to AAS	Pre-test probability of AA	Post-test probability of AA, positive test	Post-test probability of AA, negative test
US			
All patients, *n* = 497	177/497 (37%)	86/104 (83%)	91/393 (23%)
High, *n* = 52	41/52 (75%)	19/20 (95%)	22/32 (69%)
Intermediate, *n* = 258	122/258 (47%)	59/65 (91%)	63/193 (33%)
Low, *n* = 187	17/187 (9%)	8/19 (42%)	9/168 (5.4%)
CT			
All patients, *n* = 489	257/489 (53%)	253/260 (97%)	4/229 (1.8%)
High, *n* = 114	90/114 (79%)	90/91 (99%)	0/23 (0%)
Intermediate, *n* = 276	138/276 (50%)	135/148 (91%)	3/128 (2.3%)
Low, *n* = 99	16/99 (16%)	15/20 (75%)	1/79 (1.3%)

*AA*, Acute appendicitis, *AAS*, Adult Appendicitis Score

US was performed to 52 (12%) patients. Pre-test probability of appendicitis in patients that underwent US was 41 of 52 (75.0%). The post-test probability for appendicitis was for a positive US 19 of 20 (95%) and for a negative 22 of 32 (68.8%). The accuracy of US was in this group 29 of 52 (55.8%) (Tables 2-4, Fig. 2).

### Intermediate probability group (AAS 11–15)

In the group of intermediate probability of acute appendicitis there were 596 patients of whom 304 (51%) had appendicitis. CT was performed to 276 (46%) patients. Pre-test probability of appendicitis in patients that underwent CT was 138 of 276 (50%). The post-test probability for appendicitis was for a positive test 135 of 148 (91.2%) and for a negative 3 of 128 (2.3%). The accuracy of CT was in this group 260 of 276 (94.2%). (Tables 2-4, Fig. 2).

US was performed to 258 (43%) patients in the intermediate probability group. Pre-test probability of appendicitis in patients that underwent US was 122 of 258 (47.3%). The post-test probability for appendicitis was for a positive US 59 of 65 (90.8%) and for a negative 69 of 193 (32.6%). The accuracy of US was in this group 189 of 258 (73.3%). (Tables 2-4, Fig. 2).

### Low probability group (AAS ≤10)

In the group of low probability for appendicitis there were 510 patients of whom 34 (7%) had appendicitis. CT was performed to 99 (19%) patients. Pre-test probability of appendicitis in patients that underwent CT was 16 of 99 (16.2%). The post-test probability for appendicitis was for a positive CT 15 of 20 (75.0%) and for a negative 1 of 79 (1.3%). The accuracy of CT was in this group 93 of 99 (93.9%). (Tables 2-4, Fig. 2).

US was performed to 187 (37%) patients in the low probability group. Pre-test probability of appendicitis in patients that underwent US was 17 of 187 (9.1%). The post-test probability for appendicitis was for a positive US 8 of 19 (42.1%) and for a negative 9 of 168 (5.4%). The accuracy of US was in this group 167 of 187 (89.3%). (Tables 2-4, Fig. 2).

### Diagnostic performance of imaging related to prevalence of appendicitis

There was statistically significant difference between the different score groups in observed post-test probability after positive imaging result (the proportion of true positive compared to false positive imaging results). (Figure 2) In the low-probability patients, there were 15 true positive and 5 false positive CT examinations (post-test probability after positive test 75%). In the intermediate-probability patients the post-test probability was 135 of 148 (91%), and in the high-probability group 90 of 91 (99%) (*p* < 0.001, chi-square test). (Tables 3 and 4, Fig. 2).

In the low probability group, there were 8 true positive and 11 false positive US examinations and post-test probability of appendicitis after positive US was 42%. In the intermediate and high probability patients the post-test probability was 59 of 65 (90.8%) and 19 of 20 (95%), respectively (*p* < 0.001, chi-square test).

### Other diagnostic findings

In high probability group 18 (16%) patients had other specific diseases and 6 (5%) did not have diagnostic findings on CT scan. In the intermediate probability group and low probability group the rate of other diagnosis on CT were 33 and 35%, respectively. On the contrary US found other diagnosis only in 4 (7%) patients and 26 (50%) did not have diagnostic findings on US in high probability group. Other specific diagnoses were found with US in 19 (7%) and 18 (10%) patients in intermediate and low probability groups, respectively.

### Prevalence of appendicitis in patients managed without imaging

Seven hundred twenty-three (47%) patients included into prospective data collection did not undergo diagnostic imaging. Among these patients, in high probability group 261 (92.6%) out of 282 patients, in intermediate probability group 89 (51.7%) out of 172 patients, and in low probability group 6 (2.2%) out of 269 patients had appendicitis.

## Discussion

This study shows that, based on the clinical score, in patients with most improbable appendicitis (AAS ≤10), screening with US adds little benefit and can even be harmful because of considerable amount of false positive imaging results. There were more false than true positive results in US in this group, leading to a negative appendectomy rate of 58% after US in this group. When the low-probability patients underwent CT, 25% of positive results were false. In every 20 CT examinations in the low-probability group there were 3 true and 1 false positive results, leading to negative appendectomy rate of 25%. Hence only 15% of patients in low probability group had benefit from CT, whereas 85% were exposed to ionizing radiation without significant benefit in diagnosis. In the low probability group, there were no patients with perforated appendix and peritonitis. To avoid false positive imaging results and high rate of negative appendectomies, we suggest that patients with low AAS and equivocal diagnosis would undergo clinical observation instead of immediate imaging. In this group patients have vague symptoms and part of the patients probably have appendicitis that would resolve spontaneously during the follow-up [20, 21].

CT, with excellent diagnostic performance, is the best method for excluding appendicitis in the high probability patients when there is disagreement between scoring and the clinical evaluation. In the high probability group, scoring alone had in our study of the validation of AAS specificity of 93.3%, and hence we do not recommend routine imaging in this group. Also, in high probability group, patients who did not have diagnostic imaging the probability of appendicitis was 93%, which was higher than post-test probability of appendicitis after positive CT scan in intermediate probability group. However, in these patients, diagnostic performance of US is good and of CT excellent and imaging should be performed without hesitation when there is clinical suspicion of other diagnosis than appendicitis. In young patients, to avoid radiation, US should be the primary imaging modality. However, US has limited value in finding other diagnosis, and thus CT is usually needed when US is negative or inconclusive.

In meta-analysis by Parker et al. of cost and radiation savings of partial substitution of US for CT, the sensitivity and specificity of CT were 93.4 and 95.3% respectively [2]. In the meta-analysis by van Randen et al. the prevalence of appendicitis was related to post-test probability in three different populations. The analysis showed that the added value of imaging in suspected appendicitis depends on the pre-test probability of appendicitis. The respective mean sensitivity and specificity of CT were 91 and 91% [13]. In our study, the sensitivity of CT was in all patients 98.4% and specificity 97.0%. Alike in the meta-analysis by van Randen, the post-test probability after positive CT was related to the prevalence of appendicitis and differed significantly in different risk groups.

In the meta-analysis by Parker et al. the sensitivity of US was 87.5% and specificity 92.7%. In the meta-analysis by van Randen et al. mean sensitivity and specificity of US were 78 and 83% respectively. In our study the sensitivity of US in all patients was 48.6% and specificity 94.4%. In both the meta-analysis by van Randen et al. and the current study the post-test probability of appendicitis after positive US decreased dramatically along decreasing prevalence of appendicitis.

Spontaneously resolving appendicitis is a phenomenon that has been described in surgical and radiological literature [20, 22–24]. Despite the increased diagnostic accuracy of appendicitis, we are currently not able to recognize patients with resolving appendicitis in the early phase of disease. The patients with spontaneous resolution of appendicitis probably have milder, non-specific symptoms. This is supported by the studies by Decadt et al. and Morino et al. in which patients with non-specific abdominal pain were randomized to either early laparoscopy or close observation. In both studies in the laparoscopy groups, there were significantly more patients with acute appendicitis than in the observation groups [25, 26]. In suspected appendicitis, if imaging is mandatory, prevalence of uncomplicated appendicitis increases because patients with possible spontaneous resolution of appendicitis undergo surgery [27, 28]. Hence, diagnostic guidelines with conditional imaging aid to prevent surgery for patients with resolving appendicitis.

We have implemented the AAS scoring system to guide the diagnostic work-up of patients with suspected acute appendicitis. The aim of scoring is not to replace imaging. In contrary, scoring helps to avoid under- and overuse of imaging studies by targeting these investigations to patients with most equivocal diagnosis. All patients with suspected appendicitis were included in the study. Some patients, however, should be excluded from the routine diagnostic work-up. Pregnant patients should invariably undergo imaging in case of suspected appendicitis because of increased negative appendectomy rate and high risk of fetal loss after surgery [29, 30]. CT should in pregnant patients be replaced with MRI to avoid ionizing radiation [31]. Patients with clinical suspicion of appendiceal abscess should undergo CT examination. In these patients, CT, in addition to being the most accurate imaging method, also benefits in planning the treatment. In immunosuppressed patients, threshold of imaging should be low. Immunosuppression alters laboratory results and can mask the typical clinical signs and symptoms of appendicitis.

In this study, US was performed and CT reported by radiology residents and attending radiologists with varying experience. Hence this study describes well the real-life situation in the emergency setting. The preliminary reports by on-call residents are in our hospital re-evaluated next morning by a staff radiologist. However, the re-evaluation is rarely performed before the decision of treatment is made.

In the Netherlands, the national guidelines recommend mandatory imaging in suspected acute appendicitis. The primary imaging modality is US followed by CT in case of inconclusive US. With this protocol, excellent results have been published [5, 6]. In a study by Atema et al., immediate CT was compared to conditional CT after negative or non-diagnostic US [4]. The amount of CT examinations was halved with the conditional strategy, but resulted in more false positive imaging results. In our study, false positive imaging results lead to high rate of negative appendectomy in the low probability patients. We suggest that scoring would be implemented in the diagnostic work-up to exclude from the mandatory imaging protocol the low-probability patients with frequent false positive findings in imaging.

There was no cost-benefit analysis involved in this study. However, previous research suggests that mandatory imaging is cost-beneficial, and that conditional CT has cost benefits when CT is partially replaced with US [2, 5]. In the light of present study, excluding the patients with least probable appendicitis from mandatory imaging can further increase these benefits.

### Limitations

Adult Appendicitis Score is novel, and no large external validation studies have been published yet. More studies would strengthen the validation of the score. Another potential limitation of this study is that only part of patients suspected of appendicitis was imaged. Because patients underwent imaging at surgeons’ discretion, potential verification bias exists.

## Conclusions

In conclusion, this study shows that diagnostic performance of CT and US depends on pre-test probability of appendicitis. Adult Appendicitis Score (online calculator available on www. appendicitisscore.com) can be used in patients with suspected appendicitis to guide selective use of imaging studies. Patients with low probability of appendicitis according to scoring have limited value from diagnostic imaging.

## Abbreviations

AA: Acute appendicitis
AAS: Adult Appendicitis Score
CT: Computed tomography
DOR: diagnostic odds ratio
LR: negative likelihood ratio
LR+: positive likelihood ratio
MRI: Magnetic resonance imaging
US: Ultrasound
